# The zebrafish paralog *six2b* is required for early proximal pronephros morphogenesis

**DOI:** 10.1038/s41598-023-47046-3

**Published:** 2023-11-11

**Authors:** Beau Belcher, Justin Vestal, Samuel Lane, Margaret Kell, Luke Smith, Troy Camarata

**Affiliations:** 1 Biological Sciences, Arkansas State University, Jonesboro, USA; 2 Biomedical Sciences, NYIT College of Osteopathic Medicine at Arkansas State University, Jonesboro, USA; 3 Biomedical Sciences, Baptist University College of Osteopathic Medicine, Baptist Health Sciences University, 1003 Monroe Ave, Memphis, TN 38104 USA

**Keywords:** Developmental biology, Differentiation, Organogenesis

## Abstract

The transcription factor Six2 plays a crucial role in maintaining self-renewing nephron progenitor cap mesenchyme (CM) during metanephric kidney development. In mouse and human, expression at single-cell resolution has detected Six2 in cells as they leave the CM pool and differentiate. The role Six2 may play in these cells as they differentiate remains unknown. Here, we took advantage of the zebrafish pronephric kidney which forms directly from intermediate mesoderm to test *six2b* function during pronephric tubule development and differentiation. Expression of *six2b* during early zebrafish development was consistent with a role in pronephros formation. Using morpholino knock-down and CRISPR/Cas9 mutagenesis, we show a functional role for *six2b* in the development of proximal elements of the pronephros. By 48 h post-fertilization, *six2b* morphants and mutants showed disrupted pronephric tubule morphogenesis. We observed a lower-than-expected frequency of phenotypes in *six2b* stable genetic mutants suggesting compensation. Supporting this, we detected increased expression of *six2a* in *six2b* stable mutant embryos. To further confirm *six2b* function, F_0_ crispant embryos were analyzed and displayed similar phenotypes as morphants and stable mutants. Together our data suggests a conserved role for Six2 during nephrogenesis and a role in the morphogenesis of the proximal tubule.

## Introduction

The nephron is the functional unit of the kidney that is shared amongst vertebrates from skates and rays to zebrafish to turtles and mammals. Nephrogenesis in mammals occurs through reciprocal interactions between the intermediate mesoderm (IM) derived cap mesenchyme (CM), which is the nephron progenitor cell pool, and the ureteric bud (UB) epithelium^1^. A subpopulation of CM cells is induced to differentiate to give rise to the various epithelial segments of the nephron including the glomerulus, proximal tubule, and distal tubule while the collecting duct system is derived from the UB.

Six2, a member of the *sine oculis* transcription factor family, is required for nephrogenesis^2,3^. In mice and humans, Six2 marks the self-renewing CM during kidney development^4,5^. Six2 functions in the CM to maintain undifferentiated progenitor cells. Loss of Six2 results in premature differentiation, leading to renal hypodysplasia in both mouse^4,5^ and human^6^. It is thought that Six2 acts to repress differentiation signals from the UB to ensure self-renewal of the CM and prevent premature differentiation^4,5^. However, as cells leave the CM and differentiate into pre-nephron structures, such the pretubular aggregate, renal vesicle, and S-shaped body, Six2 expression is maintained suggesting a role in nephron differentiation^7–11^. In these pre-nephron structures, Six2 is restricted to areas that give rise to the proximal segments of the nephron such as the glomerulus and proximal tubule^10,11^. In addition, persistent overexpression of mouse Six2 in the CM results in cells that fail to leave the progenitor cell pool^12^. The CM cells that do form nephrons lack the differentiation markers Lhx1 and Cdh17. Therefore, Six2 may be required to suppress differentiation signals to maintain nephron progenitors while also playing a role during the early stages of nephron differentiation.

Unlike mammals where nephrons are formed from IM derived CM/UB cell interactions, the zebrafish pronephric tubules develop directly from the IM without the maintenance of a specific progenitor cell pool^13^. By 24-h post-fertilization (hpf), two epithelial tubules form, each with a glomerular rudiment at the proximal end. At this time point, some segment specific markers begin to become expressed, such as *slc20a1a* and *trpm7,* which mark the proximal convoluted and proximal straight tubule, respectively^14^. At 24 hpf, the zebrafish pronephros possesses a patent lumen and has demonstrated the secretion of fluid^15^. By 48 hpf the glomerular rudiments have fused, and the most proximal portion of the tubule begins to become convoluted with concurrent elongation of more distal nephron segments. The development of the pronephros directly from the IM allows for the direct assessment of gene function in nephron differentiation. We took advantage of this feature to determine if a zebrafish Six2 homolog plays a role in pronephric tubule morphogenesis. The zebrafish genome contains two Six2 paralogs, *six2a* and *six2b*, due to a genome duplication event during the evolution of teleosts^16^. *six2a* has previously been identified and expression during zebrafish embryogenesis partially characterized, although its role in nephrogenesis was not thoroughly characterized^6^. In the current report, we focused on the previously unstudied *six2b* during pronephros development. Using independent and complimentary approaches to disrupt *six2b* function, the transcription factor appears to be required for proper morphogenesis of proximal elements of the pronephros. This points to a highly conserved role for Six2 in the development of the nephron.

## Results

### Identification and analysis of the zebrafish Six2 paralog *six2b*

The zebrafish genome contains two Six2 paralogs, *six2a* and *six2b*, due to a genome duplication event during the evolution of teleosts^16^. The *six2a* paralog has been previously characterized^6^. Here, we cloned and characterized the *six2b* gene. Nucleotide alignment of *six2a* and *six2b* showed 43% sequence identity while the predicated amino acid sequences were 84% identical with 91% similarity (Supplemental Fig. 1). The reference sequence for *six2a* (NM_131783) was longer than the reference sequence for *six2b* (NM_001128734). The *six2a* gene sequence appears to possess longer 3′ and 5′ UTRs, similar to what is detected in mouse and human *Six2* genes (NM_011380 and NM_016932, respectively). It is not clear why the gene sequence for *six2b* is shorter than its paralog, however the predicted protein sequences only differ by three amino acids (Supplemental Fig. 1). Amino acid sequence comparison between zebrafish Six2b and human and mouse Six2 revealed 79% sequence identity with 85–86% similarity. Significant amino acid identity was observed within the Six domain of zebrafish Six2 paralogs and the mammalian homologs (Supplemental Fig. 1).

**Figure 1:**
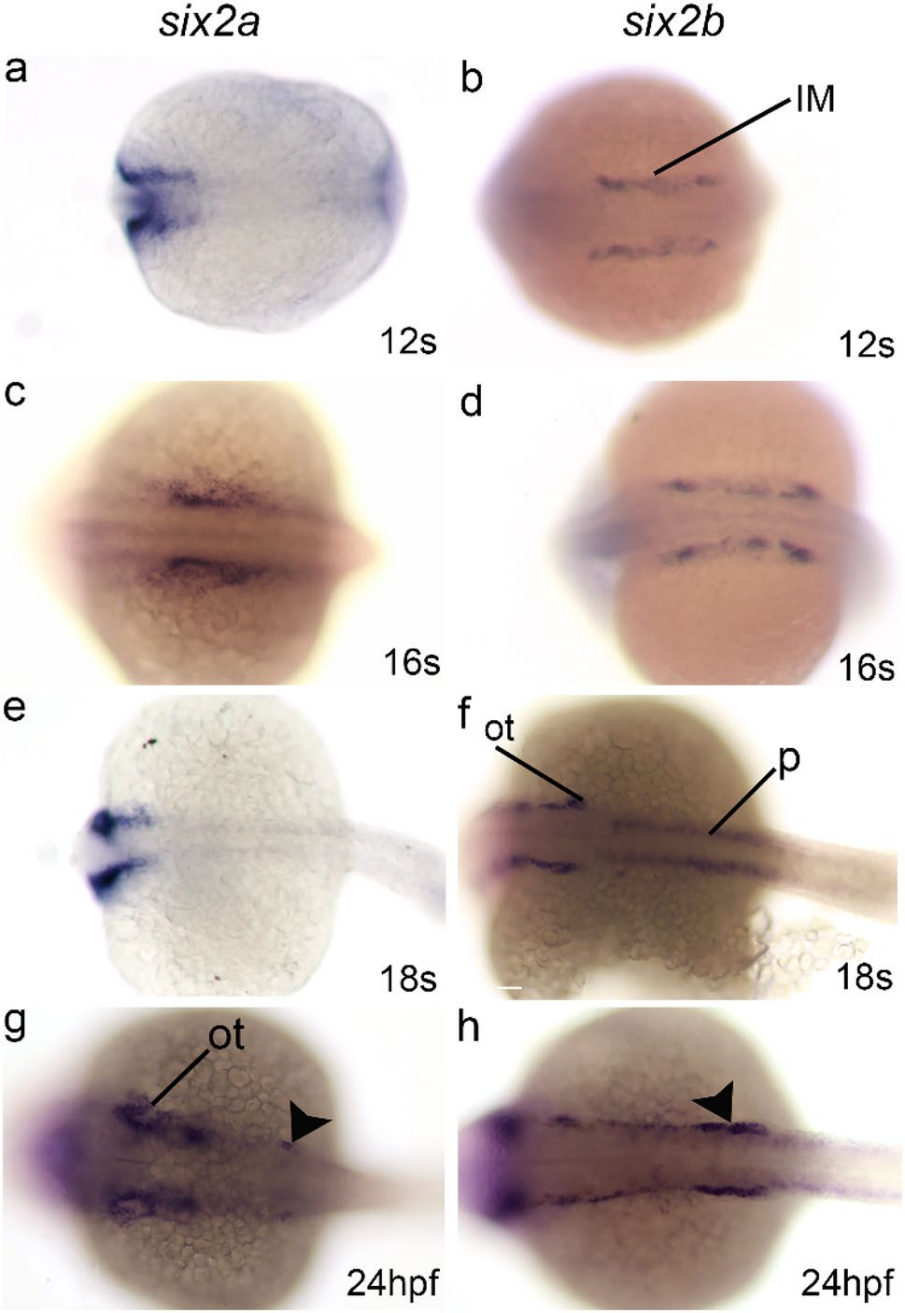
Early expression of *six2a* and *six2b* paralogs. (**a**, **c**, **e**, **g**) Whole mount *in situ* hybridization for *six2a*. Expression is detected in the developing head and otic vesicles. Focal expression was observed adjacent to the pronephric tubule at 24 hpf (arrowhead in g). (**b**, **d**, **f**, **h**) Whole mount *in situ* hybridization for *six2b*. Expression was detected in the intermediate mesoderm and otic vesicles. At 18 somites, weak signal could be detected in the pronephric tubules. By 24 hpf *six2b* was predominantly found in cells adjacent to the tubule epithelium (arrowhead in h). Head is toward the left in all images. IM, intermediate mesoderm; OT, otic vesicle; P, pronephros.

Whole mount in situ hybridization for *six2b* detected expression in the intermediate mesoderm at the 12-somite stage where it was maintained until 16 somites (Fig. 1). By 18 somites, *six2b* was clearly detected in the otic vesicles consistent with other reports (Fig. 1f)^17^. Expression of *six2b* was also detected in the proximal portion of the pronephric tubules (Fig. 1f). In contrast, *six2a* was predominantly detected in the otic vesicles with transient expression in the intermediate mesoderm (Fig. 1)^6^. By 24 h-post-fertilization (hpf), both *six2a* and *six2b* expression was restricted to cells adjacent to the proximal pronephric tubule (Fig. 1g,h). After 24 hpf *six2a* and *six2b* expression was restricted to otic vesicles and other structures in the head with no detectable expression within the pronephros. The expression pattern of *six2b* within the IM and pronephric field is consistent with a role in nephrogenesis.

### Knock-down of *six2b* results in pronephric tubule defects

**Figure 2:**
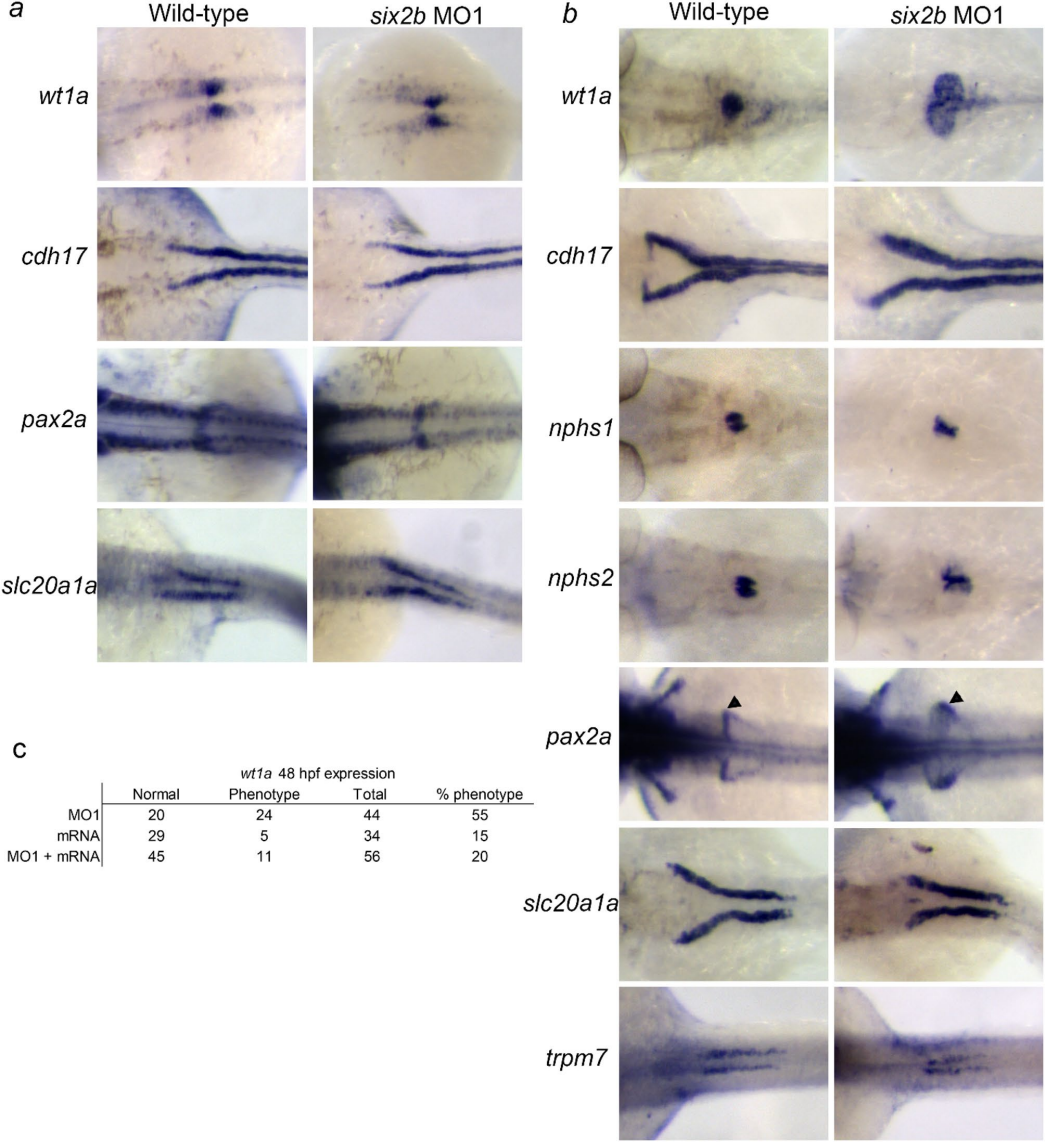
Pronephric marker expression following injection of *six2b* MO1. (**a**) Expression of pronephric markers at 24 hpf following MO1 injection. No significant changes in gene expression or tissue morphology were observed. (**b**) Expression of pronephric markers at 48 hpf following MO1 injection. Knock-down of *six2b* resulted in glomerular defects as seen with *wt1a*, *nphs1*, and *nphs2*. Proximal tubule morphogenesis was defective using *cdh17* and *slc20a1a* probes. The proximal straight tubule appeared unaffected using *trpm7*. (**c**) Compiled data of *wt1a* morphological defects following mouse *Six2* mRNA injection alone or combined with MO1. *Six2* mRNA co-injection partially rescued the MO1 induced *wt1a* phenotype. Arrowheads for 48 hpf *pax2a* expression denote neck region.

To gain insight into whether *six2b* plays a role in zebrafish pronephric tubule development, we used antisense morpholino oligonucleotides (MO) to knock-down *six2b* expression. A previous study using an ATG targeted MO to knock-down *six2a* resulted in non-specific early embryonic dorsoventral patterning defects^6^. Injection of a *six2b* ATG targeting MO resulted in similar non-specific dorsalization of early embryos preventing analysis of the pronephros^18^. We then designed a *six2b* MO targeting the splice junction between exon 1 and intron 1 (Supplemental Fig. 2). Injection of the splice inhibiting MO (hereafter referred to as MO1) resulted in a smaller transcript as detected by PCR and confirmed by sequencing (Supplemental Fig. 2). Injection of MO1 induced the usage of a cryptic splice site within exon 1 resulting in a 59-nucleotide deletion in the mRNA, altering the reading frame. The predicted amino acid sequence of Six2b induced by MO1 removed a portion of the DNA binding homeodomain and the entire C-terminus (Supplemental Fig. 2c). Injection of *six2b* MO1 did not result in early embryo dorsalization defects (Fig. 2; Supplemental Fig. 2d), and therefore was used to investigate the role of *six2b* in pronephric tubule development.

One-cell stage embryos were injected with 4 ng MO1, and pronephric tubule development was evaluated at 24 and 48 hpf (Fig. 2; Table 1). At 24 hpf, no dramatic phenotypic changes were observed between uninjected controls and MO1 injected embryos (Fig. 2a; Table 1). Expression of the glomerular marker *wt1a* and epithelial marker *cdh17* appeared normal in knock-down embryos. Similarly, no changes in expression at 24 hpf were found for either *pax2a*, a marker of the neck region, or *slc20a1a*, which marks the proximal convoluted tubule (PTC; Fig. 2a). However, at 48 hpf, proximal tubule defects were apparent in *six2b* knock-down embryos (Fig. 2b; Table 1). At this stage of development, a single glomerulus can be detected in wild-type embryos by the expression of *wt1a*. In MO1 injected embryos, *wt1a* expression revealed two expanded cyst-like domains. In wild-type embryos at 48 hpf, *cdh17* remained expressed throughout the pronephric epithelium and revealed the initiation of proximal convolution (Fig. 2b). This proximal tubule morphology was disrupted in close to half of *six2b* knock-down embryos (Fig. 2b; Table 1). Proximal tubule morphological defects could be detected further using markers for the neck region, podocytes, and proximal convoluted tubule (Fig. 2; Table 1). The expected expression domains for *pax2a*, *nephrin (nphs1)*, *podocin (nphs2)*, and *slc20a1a* were disrupted at 48 hpf following MO1 injection. A marker for the proximal straight tubule (PST), *trpm7*, showed normal expression in MO1 injected embryos suggesting knock-down of *six2b* only disrupted more proximal elements of the pronephros. To determine the efficacy of *six2b* MO1, a dilution series was conducted as well as a co-injection of MO1 with *Six2* mRNA (Supplemental Fig. 2; Fig. 2c)^19^. Using *wt1a* expression at 48 hpf as a readout, increased percentages of glomerular phenotypes were detected with increasing dosage of MO1 (Supplemental Fig. 2). Co-injection of MO1 with 50 pg of mouse *Six2* mRNA was able to partially rescue the MO1 induced *wt1a* phenotype at 48 hpf (Fig. 2c; *p* < 0.001). Taken together, this data suggests that *six2b* function is required for appropriate morphogenesis of the more proximal elements of the pronephros.

**Table 1:** s*ix2b* MO1 injection percent phenotype (total N).

Probe	24 hpf		48 hpf		
	Wild-type	MO1	Wild-type	MO1	Fisher's *p*-value 48 hpf
*wt1a*	0 (48)	0 (40)	0 (98)	55 (44)	< 0.0001
*cdh17*	0 (41)	0 (49)	0 (87)	49 (37)	< 0.0001
*slc20a1a*	0 (57)	0 (55)	0 (31)	50 (44)	< 0.0001
*pax2a*	0 (54)	0 (51)	0 (33)	38 (53)	< 0.0001
*nphs1*	ND	ND	0 (53)	33 (30)	< 0.0001
*nphs2*	ND	ND	0 (38)	33 (33)	< 0.0001
*trpm7*	ND	ND	0 (83)	0 (36)	ND

### Stable CRISPR/Cas9 mutagenesis of *six2b* results in limited proximal tubule phenotypes

**Figure 3:**
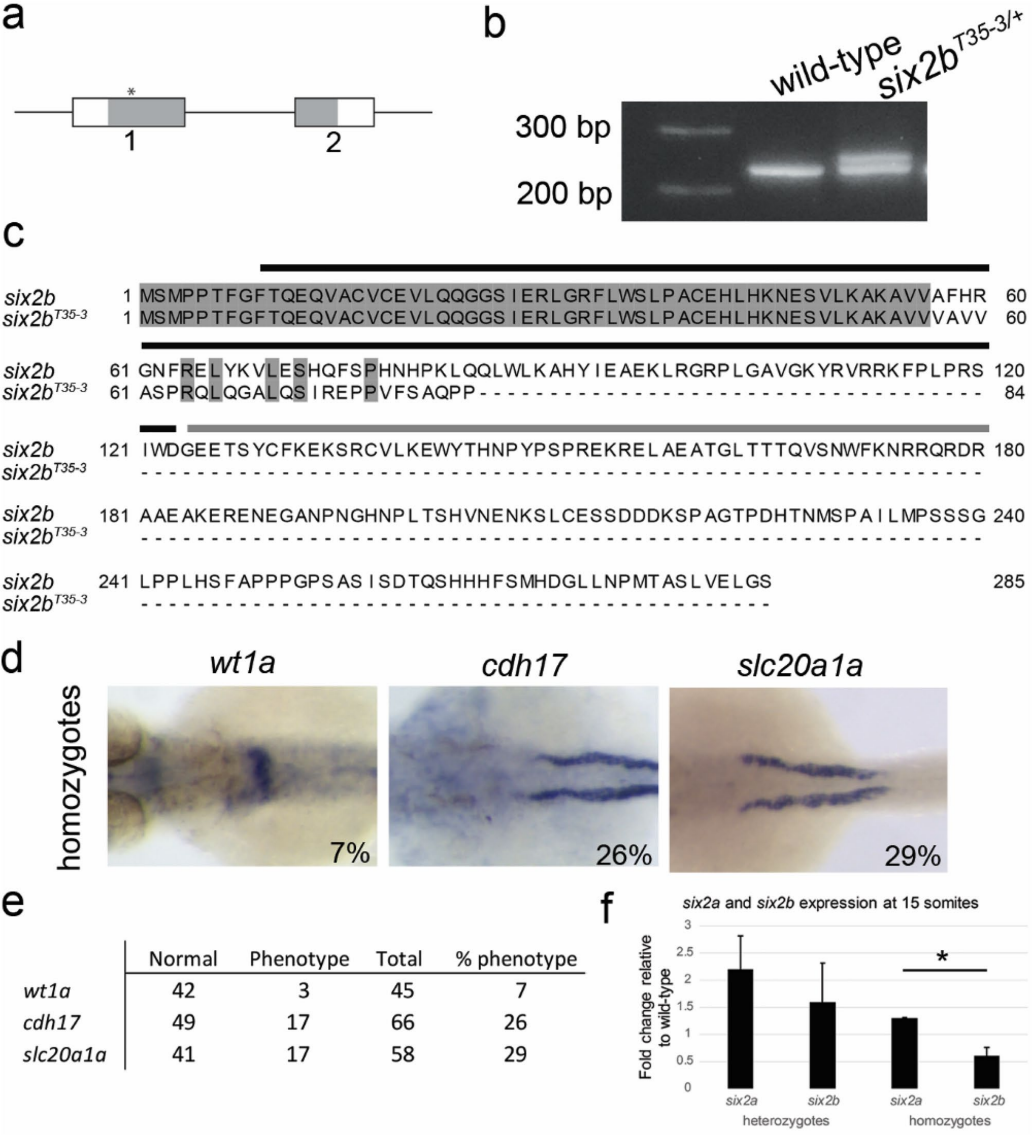
Stable CRISPR/Cas9 mutagenesis of *six2b*. (**a**) Schematic of mutagenesis target site. (**b**) PCR analysis of stable line *six2b*^*T35-3*^ genotype differentiating between heterozygous and wild-type animals. (**c**) Amino acid alignment of wild-type and mutant Six2b. The mutant line is predicted to lose most of the Six domain and the entire homeodomain and C-terminus. Black bar covers Six domain, grey bar covers homeodomain. (**d**) Whole mount in situ hybridization of homozygous mutant embryos at 48 hpf for *wt1a*, *cdh17*, and *slc20a1a*. Proximal tubule defects were detected in homozygous embryos. (**e**) Compiled data of proximal tubule morphology defects observed in *six2b* homozygous mutants. For comparison, heterozygous embryos are displayed in Supplemental Fig. 3 (**f**) qPCR of *six2a* and *six2b* in heterozygous and homozygous *six2b* mutants at 15 somites. Expression is relative to wild-type embryos at the same developmental stage. Error bars are standard error of the mean. *p-value = 0.03.

To confirm the phenotypes observed by morpholino knock-down, we established a stable mutant line using CRISPR/Cas9 mutagenesis (see Methods). A target in *six2b* exon 1, near the beginning of the coding region, was used for guide RNA design and mutagenesis (Fig. 3a). One founder line (*six2b*^*T35-3*^) was identified from allele sequencing to contain an altered *six2b* genomic sequence at the CRISPR target site (Supplemental Fig. 3a). Sequence analysis suggested five nucleotides of original DNA were deleted and repaired with a 15 nucleotide insertion, which resulted in a premature stop codon within the Six protein–protein interaction domain (Fig. 3b,c). Consequently, the predicted protein was expected to be missing close to half of the Six domain and the entire homeodomain and C-terminus. Therefore, we predicted this mutation would behave as a loss-of-function allele. The stable fish line was outcrossed to wild-type fish for five generations to remove off-target CRISPR/Cas9 sites and maintained as heterozygous mutants. Initial in-crossing of *six2b*^*T35-3/*+^ heterozygotes yielded expected Mendelian genotype ratios, however, expected percentages of phenotypes for *wt1a* and *cdh17*, as observed for MO1, were not detected (Supplemental Fig. 3). We next created a homozygous line, *six2b*^*T35-3/T35-3*^, and reassessed pronephric tubule marker expression at 48 hpf (Fig. 3d,e). Expression of the glomerular marker, *wt1a*, was predominantly normal in homozygous embryos. However, a straight proximal tubule phenotype was observed using *cdh17* and *slc20a1a* gene markers in 26% and 29% of homozygous embryos, respectively (*p* < 0.001; Fig. 3d,e).

Genetic compensation has been found in zebrafish CRISPR/Cas9 induced mutations that lead to premature stop codons^20–22^. It is possible that the reduction in the expected frequency of pronephric tubule defects in *six2b*^*T35-3/T35-3*^ embryos could be due to mechanisms of genetic compensation. To begin to investigate this, we injected *six2b*^*T35-3/T35-3*^ homozygous embryos with MO1 and assessed *wt1a* expression at 48 hpf with the expectation that *six2b* mutants would be resistant to MO knockdown. Of the MO1 injected homozygous mutant embryos, 22% (9/41) displayed a *wt1a* glomerular phenotype. This was significantly lower than MO1 injected wild-type embryos (Fig. 2c; *p* = 0.004). The paralog *six2a* could contribute to compensation in the *six2b* stable mutant line. To investigate this, we determined relative gene expression of *six2a* and *six2b* in heterozygous and homozygous embryos at 15-somites, a stage both were found to be robustly expressed (see Fig. 1). In heterozygous embryos, the expression of both paralogs was increased compared to wild-type with *six2a* displaying a greater level of overexpression (Fig. 3f). In homozygous embryos, *six2b* was greatly reduced while *six2a* exhibited an expression level approximately 20% over wild-type levels (Fig. 3f). These results were supported by our inability to clone full-length *six2b* from homozygous mutants while full-length *six2a* was detected. Together, this data supports a role of *six2b* in the morphogenesis of the pronephric proximal tubule, however, genetic compensation may be responsible for the reduced frequency of phenotypes in the stable *six2b*^*T35-3/T35-3*^ mutant.

### F_0_ CRISPR/Cas9 mutagenesis recapitulates knock-down and stable mutant phenotypes

**Figure 4:**
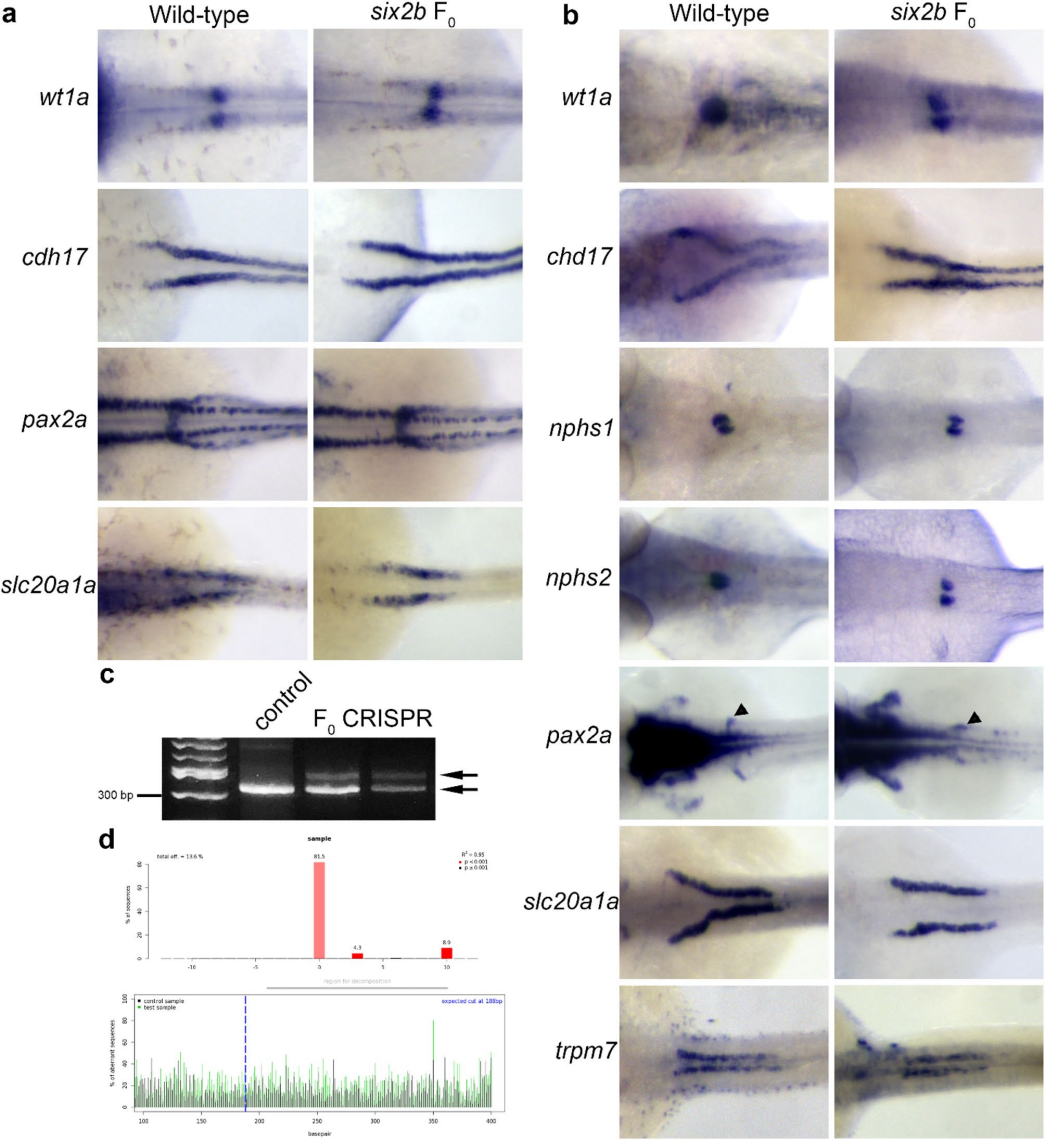
Pronephric marker expression in F_0_ embryos following *six2b* CRISPR/Cas9 reaction mix injection. (**a**) Pronephric marker expression at 24 hpf. No significant changes in gene expression or tissue morphology were observed. (**b**) Expression of pronephric markers at 48 hpf. Morphological defects in the glomerulus and proximal tubule are observed following genetic disruption of the *six2b* locus. The proximal straight tubule marked by *trpm7* appeared unaffected. Arrowhead for *pax2a* denotes the neck region. (**c**) PCR detection of genomic DNA lesions in the *six2b* gene from two independently injected embryos displaying defective *wt1a* expression domain morphology. Bottom arrow points to wild-type allele amplicon. (**d**) Representative TIDE analysis of F_0_ CRISPR/Cas9 injected embryo following in situ hybridization for *wt1a*.

To provide further support that *six2b* function is involved in proximal tubule morphogenesis, we analyzed F_0_ embryos injected with the same CRISPR/Cas9 target mix used to create the stable mutant line. Pronephros marker gene expression was determined at 24 hpf and 48 hpf following CRISPR/Cas9 injection (Fig. 4). At 24 hpf, no alterations in marker gene expression were observed, similar to MO1 injected embryos (Fig. 4a; Table 2). By 48 hpf, defects in the proximal pronephros were detected. In F_0_ crispants at 48 hpf, *wt1a* expression was observed in two separate domains similar to what is normally detected at 24 hpf (Fig. 4b; Table 2). Two separate glomerular fields were also found with the podocyte markers *nphs1* and *nphs2*. The normal positioning of the *pax2a* neck region was disrupted in F_0_ crispants and appeared similar to what was detected at 24 hpf. At 48 hpf, there was an observed straight tubule phenotype in crispant embryos (Fig. 4b; Table 2). Both *cdh17* and *slc20a1a* markers revealed reduced curvature normally observed by this time in the PCT. The PST, as marked by *trpm7* expression, was not affected. To confirm the CRISPR/Cas9 injection mix was inducing DNA lesions in the F_0_ embryos, PCR was used to amplify the target region in *six2b* from embryos post in situ hybridization that displayed a *wt1a* phenotype. Multiple PCR amplicons could be detected by agarose gel electrophoresis from F_0_ injected *wt1a* phenotypic embryos, confirming the genomic disruption of *six2b* (Fig. 4c). F_0_ injected embryos were expected to be mosaic for genetic lesions and this was confirmed using TIDE analysis of sequence reads from post in situ hybridization PCR (Fig. 4d)^23^ Collectively, the data supports a role for *six2b* in the morphogenesis of the zebrafish proximal tubule.

**Table 2:** *six2b* F_0_ crispr target injection percent phenotype (total N).

Probe	24 hpf		48 hpf		
	Wild-type	24 hpf	Wild-type	48 hpf	Fisher's *p*-value 48 hpf
*wt1a*	0 (20)	0 (87)	0 (41)	44 (64)	< 0.0001
*cdh17*	0 (42)	0 (81)	0 (40)	53 (62)	< 0.0001
*slc20a1a*	0 (21)	0 (63)	0 (35)	55 (53)	< 0.0001
*pax2a*	0 (21)	0 (83)	0 (33)	51 (86)	< 0.0001
*nphs1*	ND	ND	0 (40)	54 (67)	< 0.0001
*nphs2*	ND	ND	0 (33)	60 (57)	< 0.0001
*trpm7*	ND	ND	0 (21)	1 (69)	0.45

## Discussion

Here we aimed to determine if the zebrafish Six2 paralog, *six2b,* played a role in the development of the pronephric epithelium. Using multiple independent approaches, including morpholino knockdown along with stable and transient CRISPR/Cas9 genetic disruption, we show that appropriate morphogenesis of the proximal tubule is dependent on *six2b* function. The proximal defects following disruption of *six2b* are consistent with the expression of the transcription factor during nephrogenesis. Mammalian Six2 is detected in cells of pre-nephron structures that will give rise to proximal elements such as the glomerulus and PCT^10,11^.

The lower-than-expected frequency of observed phenotypes in the *six2b* homozygous mutant could suggest genetic compensation. Both zebrafish Six2 paralogs showed moderate upregulation in heterozygous embryos while only *six2a* was upregulated in *six2b*^*T35-3/T35-3*^ mutants. It is possible that *six2a* is partially compensating for loss of *six2b*; however, we cannot rule out other factors may be involved. Inconsistent phenotypes have been identified when comparing morphants and genetic mutants^24,25^. Some of these discrepancies are due to genetic compensation, especially from mutations that cause premature stop codons, similar to what was created through our CRISPR/Cas9 mutagenesis approach of *six2b*^21,22,26^. The observed proximal tubule phenotypes are relatively consistent between the stable and transient mutagenesis, and morpholino knockdown. This supports a hypothesis involving genetic compensation and not off-target effects.

Morphological defects were found in the glomerulus and proximal tubule following *six2b* knock-down or genetic disruption. Glomerular phenotypes were slightly different between morphants and CRISPR/Cas9 injected embryos. Both approaches resulted in a lack of glomerular field fusion at 48 hpf; however, the morpholino commonly produced a cystic glomerular phenotype which was not detected in F_0_ crispant embryos. This difference may be due to the nature of the *six2b* disruption with the genetic lesion causing loss-of-function mutations while the change in mRNA from the splice-inhibiting MO1 may induce a dominant negative form of Six2b. Sequencing of *six2b* cDNA from splice-inhibiting MO1 injected embryos predicted a protein that retained the Six protein–protein interaction domain but disrupted the DNA binding homeodomain. This could allow Six2b to bind protein partners but be unable to interact with DNA target sites to impact transcription. Podocyte morphology defects were also present in *six2b* disrupted embryos using markers *nphs1* and *nphs2*. Similar phenotypes of either reduced podocyte marker expression domains or lack of glomerular fusion have been observed following morpholino knockdown of *wt1b*^27^ as well as from morpholino or genetic disruption of *osr1*^28–30^. Common nephrogenesis related phenotypes in zebrafish involving disruption of *six2b*, *wt1a/b*, and *osr1* are to be expected. In the mouse, nephrogenesis and nephron progenitor cap mesenchyme (CM) maintenance is dependent upon Six2, Wt1, and Osr1 function^31,32^. Six2 was shown to co-immunoprecipitate with Osr1 and both genes acted synergistically during kidney development^31^. Additionally, Wt1 and Osr1 proteins interact to properly specify CM populations while Wt1 and Six2 interact to maintain the proper transcriptional programs needed for nephrogenesis^8,32^. A *Six1/2-2*, *Osr* gene pathway has even been identified in planarian protonephridia development and regeneration^33^. The similar phenotypes in the zebrafish pronephros from disruption of *wt1a/b*, *osr1*, and *six2b,* along with complementary data from the mouse model suggests this pathway of transcription factors has been highly conserved for nephron development.

The consistent phenotype observed with all three *six2b* perturbation approaches was the presence of a straight tubule at the most proximal portion of the pronephros. At 48 hpf, the beginning of proximal tubule convolution can be detected. When *six2b* was disrupted using MO1 or CRISPR mutagenesis, the most proximal portion of the pronephric epithelium remained as two parallel tubules and did not display the expected morphology as detected by *cdh17* or *slc20a1a* gene markers. This tubule morphology is comparable to what is normally observed in embryos at 24 hpf. Similarly, *pax2a* expression at 48 hpf in *six2b* knock-down or knock-out embryos was very similar to the neck region morphology in 24 hpf embryos. Furthermore, our *six2b* disruption approaches did not impact tubule formation at 24 hpf, suggesting the morphological defect is restricted to between 24 and 48 hpf. One of the major processes during this time is collective cell migration toward the proximal pole of the nephron^34,35^. An increase in cell migration rate toward the proximal pole of the nephron has been shown to start by 28.5 hpf^34^. The increase in tubule cell migration corresponds with the onset of fluid flow from the glomerulus. Disruption of flow down the pronephric tubules from either knock-down of the cardiac protein *tnnt2* or from physical obstruction caused a loss of proximal migration and convolution^34^.

Interestingly, obstruction of pronephric tubules or disrupting tubule flow by interfering with cilia function via *ift88* knock-down did not prevent glomerular field fusion^34^. It may be that the possible failure of collective cell migration in *six2b* disrupted embryos is secondary to loss of fluid flow due to improper glomerular establishment. Loss of podocytes have been detected in *osr1* morphants and genetic mutants^28,30^. Embryos with reduced *osr1* expression have a straight proximal tubule phenotype, similar to the *six2b* disruption induced phenotype^28^. In the absence of *osr1* function, pronephros progenitor cells are established but then undergo apoptosis resulting in loss of glomerular structure^30^. Reduced glomerular fusion and podocytes in *six2b* disrupted embryos may be due to an imbalance between proliferation and apoptosis. The Six family of transcription factors play critical roles in regulating cell proliferation and balancing with apoptosis^3^. Mammalian Six2 has been found to occupy the promoter of *Ccnd1* (Cyclin D1) in nephron progenitor cells^8^. It may be that *six2b* helps promote proliferation and protect against apoptosis in glomerular progenitor cells of the zebrafish pronephros. Without the Six2b transcription factor, glomerular progenitor cells are lost, resulting in a lack of fluid filtration and flow through the nephron epithelium. The loss of fluid flow would then lead to reduced collective cell migration resulting in the observed failure of proximal tubule convolution. Our results from both morpholino and genetic mutagenesis support a conserved role for *six2b* in nephron morphogenesis.

## Methods

### Zebrafish

This study was carried out in accordance with protocols approved by the NYIT College of Osteopathic Medicine and Arkansas State University Institutional Animal Care and Use Committee. Wild-type AB fish were obtained from the Zebrafish International Resource Center (ZIRC). Embryos were cultured in 0.0045% phenylthiourea (PTU) beginning between 22 and 24 h post-fertilization (hpf) to inhibit pigmentation and were staged according to Kimmel et al.^15^.

### Cloning of six2b

Full-length zebrafish *six2b* was cloned using primers based on the Genbank sequence NM_001128734. Nested primers were used for *six2b* isolation; outer forward 5′ gtggatttcgccttcaaaccaacg 3′, outer reverse 5′ tccttatgatccaagttccaccag 3′, nested forward 5′ cacttagcaatgtctatgccaccaac 3′, nested reverse 5′ cttatgatccaagttccaccaggc 3′. Full-length *six2a* was cloned using outer forward 5′ gacatacaagtacaaagagggacg 3′, outer reverse 5′ ccatgtctatgcttccaacattcg 3′, and nested forward 5′ gggtccacgtttaagagccaagg 3′, nested reverse 5′ tccacgtttaagagccaaggtcg 3′ based on Genbank sequence NM_131783. Nucleotide and amino acid alignments were conducted using Jalview^36^.

### CRISPR/Cas9 mutagenesis

CRISPR/Cas9 target identification and sgRNA template were produced using protocols described by Gagnon et al.^37^. The webtool CHOPCHOP was used to identify mutagenesis targets sites, sgRNA, and PCR primers (Supplemental Table 1)^38^. Cas9 protein was obtained from Integrated DNA Technologies (product # 1081058). CRISPR/Cas9 injection mix was determined using CrispantCal webtool as described by Burger et al.^39^ One-cell stage embryos were injected with CRISPR/Cas9 reaction mix targeting a site in *six2b* exon 1 (Fig. 3). To create a stable mutant, injected embryos were raised to adulthood and outcrossed to the AB wild-type strain. Embryos from F_1_ outcrosses were screened for indels as described by Gagnon et al.^37^ to identify founders. Genomic DNA fragments including the mutagenesis site were amplified by PCR and sequenced. One fish line was identified that induced a premature stop codon (Fig. 3) and was designated *six2b*^*T35-3*^. This fish line was outcrossed to AB wild-type animals for five generations. The stable line was maintained as either a heterozygous or homozygous strain for experimentation. For F_0_ analysis, one-cell stage embryos were injected with CRISPR/Cas9 injection mix targeting the same site within *six2b* and embryos were fixed at appropriate time points with 4% formaldehyde prepared from paraformaldehyde. For sgRNA and PCR primer sequences see Supplemental Table 1.

### Morpholino and mRNA injection

Antisense morpholino (MO) oligonucleotides were obtained from Gene Tools (Gene Tools, LLC). *six2b* MO1 was targeted to the splice donor site of exon 1 (Supplemental Fig. 2). Embryos were injected at the one-cell stage and fixed at appropriate time points with 4% formaldehyde prepared from paraformaldehyde. Uninjected sibling embryos were fixed along with morpholino injected embryos as controls. For MO1 knock-down experiments, 4 ng of morpholino was injected into each embryo unless otherwise stated. MO1 sequence 5′ gcctcaataagttctcctacctttc 3′. Translational start site MO targeting *six2b* was 5′ caaaagttggtggcatagacattgc 3′.

Mouse *Six2* mRNA was synthesized using the mMessage mMachine kit (Ambion) as described^40^. Embryos were injected with 50 pg of *Six*2 mRNA at the one-cell stage and fixed at appropriate time points with 4% formaldehyde prepared from paraformaldehyde. Uninjected sibling embryos were fixed along with mRNA injected embryos as controls. The amount of mRNA injected was determined by dilution series at the concentration that produced the lowest toxicity.

### In situ* hybridization*

Whole mount in situ hybridization was performed as previously described^41^. Sequenced *six2a* and *six2b* cDNA was cloned into a GATEWAY (Life Technologies) compatible pBluescriptII KS + vector for antisense probe production. Antisense RNA was produced from linearized vector using T3 RNA polymerase (New England Biolabs). Pronephric tubule markers *wt1a*, *pax2a*, *cdh17*, *slc20a1a*, *nphs1*, and *nphs2* were obtained from the Drummond lab.

### RNA extraction and qPCR

Total RNA was extracted from embryos using the Monarch Total RNA miniprep kit with on-column DNase I treatment following manufacturer’s instructions (New England Biolabs). Total RNA concentration was determined by Nanodrop (Fisher Scientific). Reverse transcription was used to create cDNA that was used in qPCR assays. CFX96 real-time PCR detection system was used to run qPCR experiments with iTaq Universal SYBR green supermix (Bio-Rad). qPCR runs were performed with three technical replicates per run and conducted with four independent runs. Relative fold differences in gene expression were determined using the ΔΔCT method. Expression was normalized using *ef1α*. *six2a* forward 5′ gcgaaggagagggagaataatg 3′, reverse 5′ acgaggtgtgatcaggagta 3′; *six2b* forward 5′ gagagcgtattgaaagcgaaag 3′, reverse 5′ gttgaagtttagggtggttgtg 3′; *ef1α* forward 5′ ctggaggccagctcaaacat 3′, reverse 5′ atcaagaagagtagtaccgctagcattac 3′.

### Genomic DNA extraction and PCR

Genomic DNA was extracted from single embryos or fin clips using Monarch Genomic DNA purification kit (New England Biolabs). Genomic DNA was used for PCR analysis and sequencing to identify the presence of *six2b* indels following protocols described by Gagnon et al.^37^. Primer sequences are listed in Supplemental Table 1. For sequencing, a forward primer containing a T7 sequencing site was utilized. PCR amplicons were purified using the Monarch PCR and DNA cleanup kit (New England Biolabs) and submitted for sequencing. Sequence reads from F_0_ CRISPR/Ca9 injected embryos was analyzed using TIDE program^23^.

### Statistics

Comparisons between control and experimental groups post in situ hybridization were conducted using Fisher’s exact test. To determine Mendelian ratios of mutant genotypes, Chi square test was performed. Relative gene expression comparisons were conducted using Mann–Whitney U-test as described^42^. The threshold for statistical significance was set at 0.05.

### Supplementary Information

Supplementary Information 1.

Supplementary Information 2.

## Data Availability

The reagents generated during the current study are available from the corresponding author on reasonable request.
