# Supplementary material for: The zebrafish paralog six2b is required for early proximal pronephros morphogenesis
Source: Sci Rep. 2023 Nov 11;13:19699. doi: 10.1038/s41598-023-47046-3 (PMC10640633; doi:10.1038/s41598-023-47046-3)
Supplement: Supplementary file 1 — Supplementary Information 1. [file 41598_2023_47046_MOESM1_ESM.pdf]

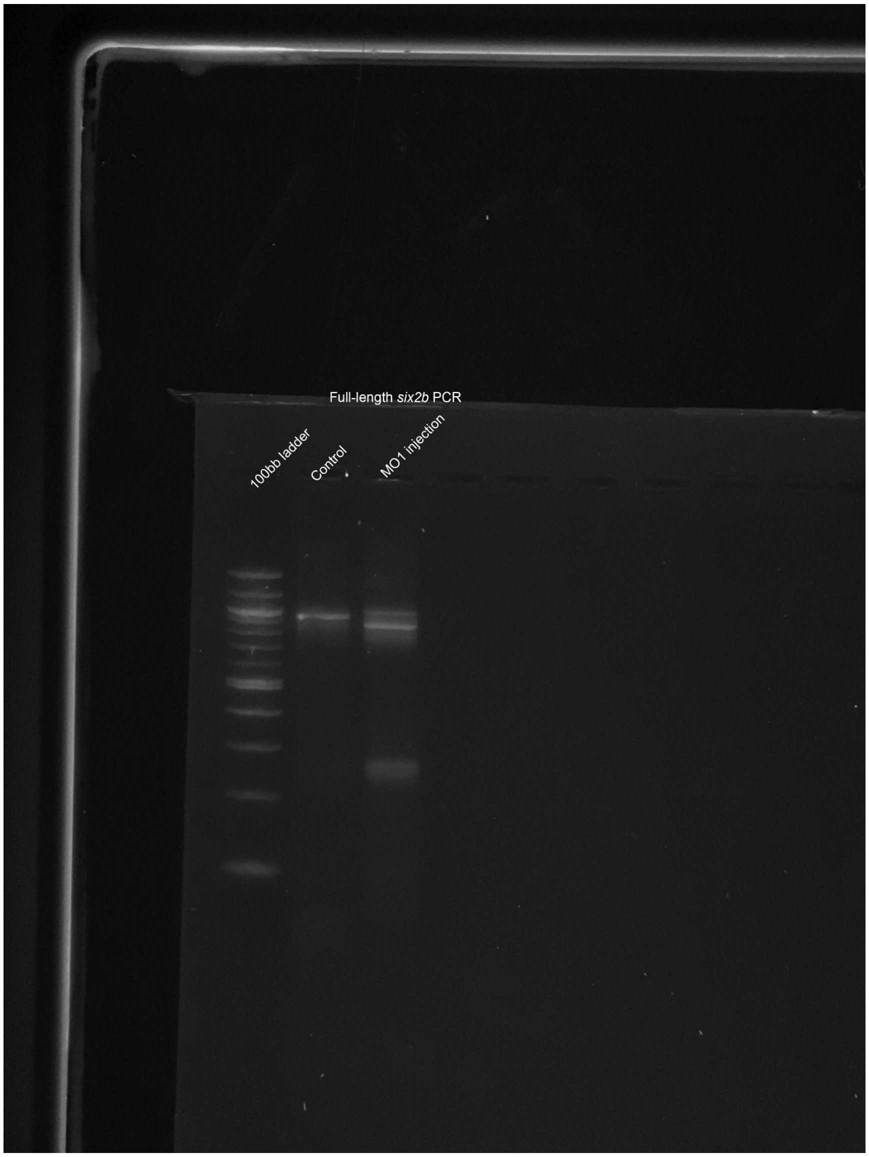

Supplemental data: Full agarose gel for *six2b* full-length PCR comparing wild-type control and MO1 induced deletion.

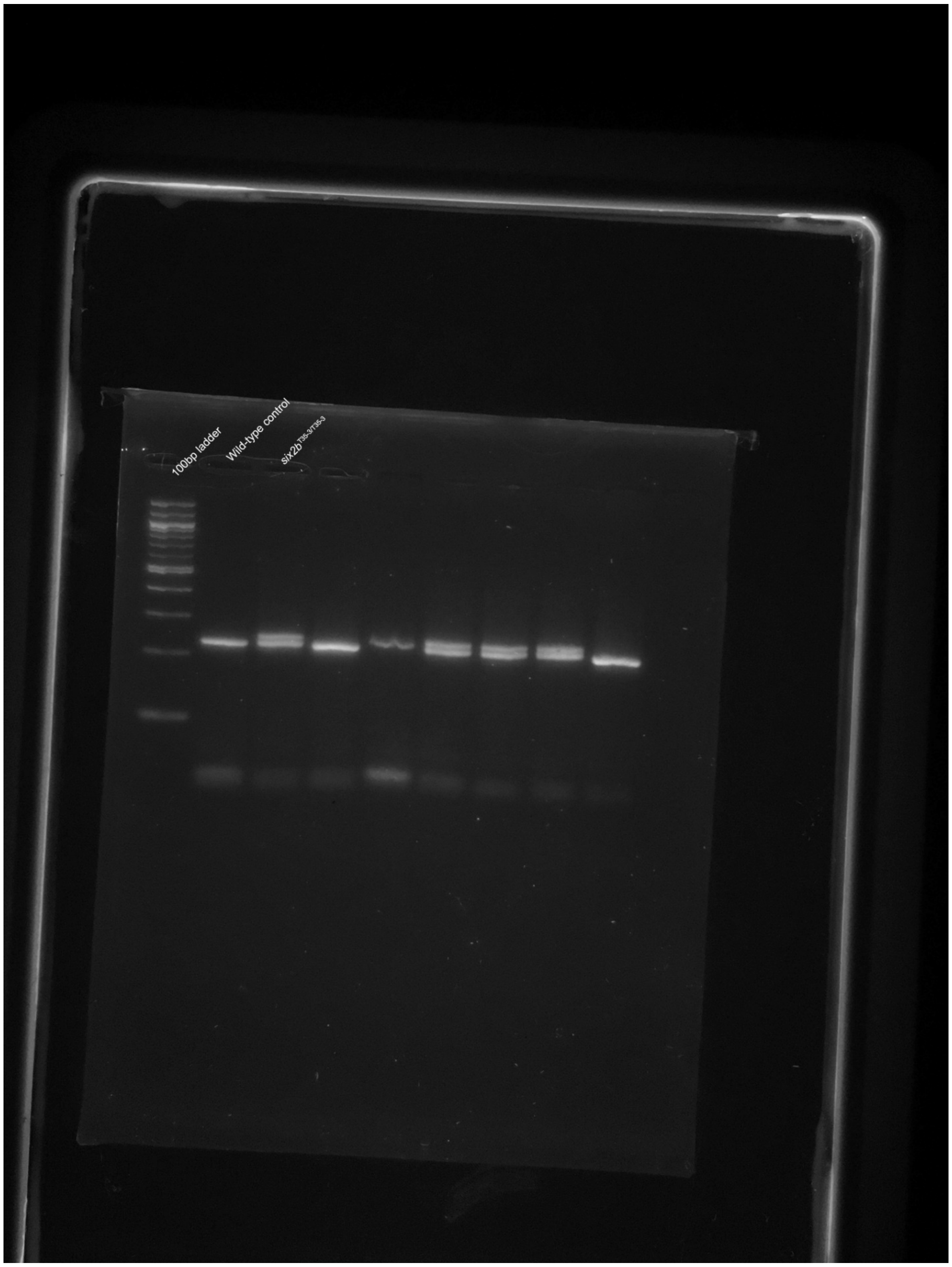

Supplemental data: Full gel of CRISPR/Cas9 target PCR comparing wild-type *six2b* and heterozygous *six2b*$^{T35-3}$.

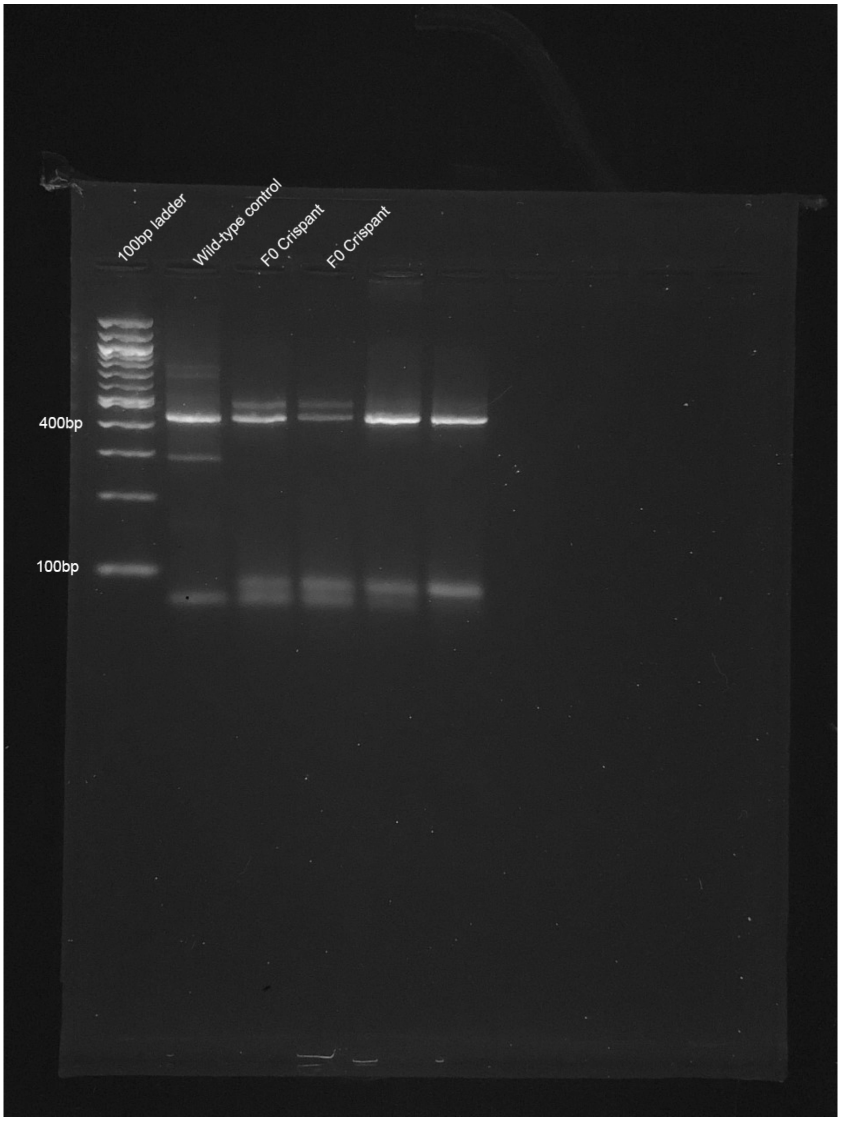

Supplemental data: Full gel of *six2b* CRISPR/Cas9 F_0 injection following embryo *in situ* hybridization. Wild-type control is PCR from embryo with normal *wt1a* expression domain. F_0 crispant lanes are from independent embryos with *wt1a* phenotypes.
