# Supplementary material for: The zebrafish paralog six2b is required for early proximal pronephros morphogenesis
Source: Sci Rep. 2023 Nov 11;13:19699. doi: 10.1038/s41598-023-47046-3 (PMC10640633; doi:10.1038/s41598-023-47046-3)
Supplement: Supplementary file 2 — Supplementary Information 2. [file 41598_2023_47046_MOESM2_ESM.pdf]

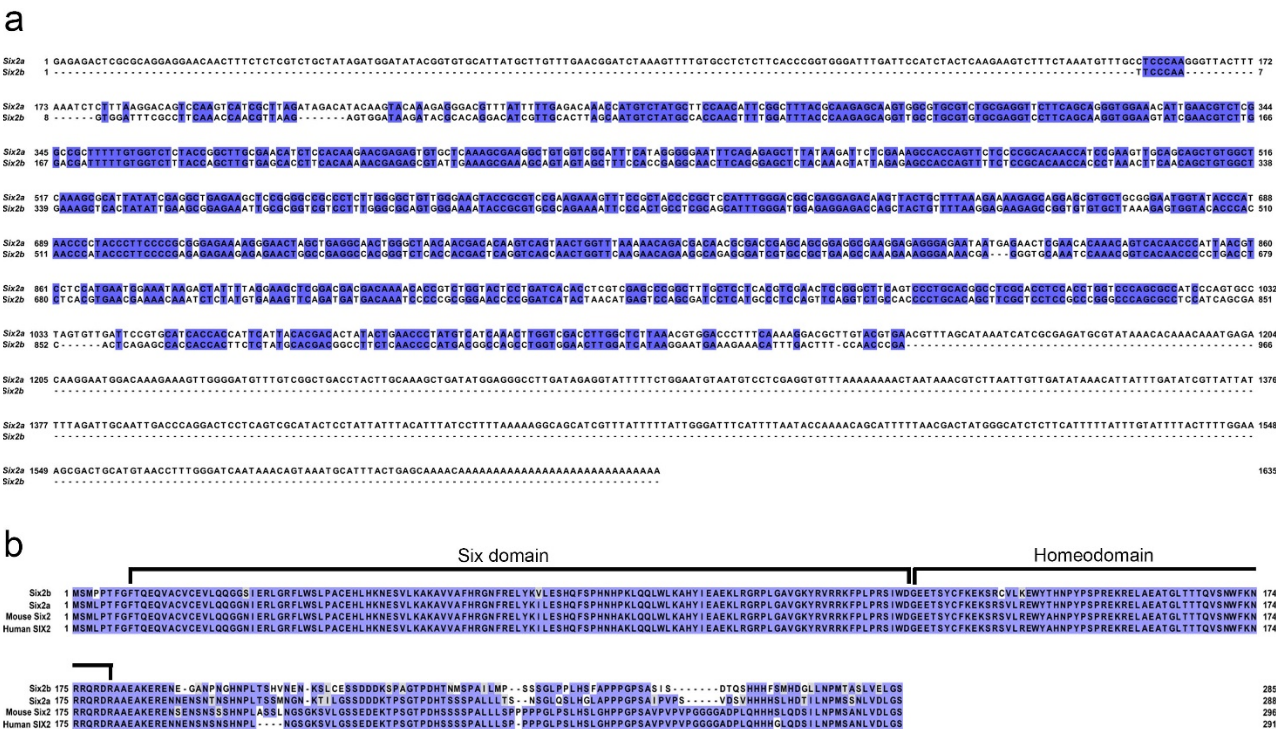

Supplemental Figure 1: Nucleotide and amino acid alignments of zebrafish Six2a and Six2b. (a) Nucleotide gene alignment of *six2a* and *six2b*. The paralogs share 43% gene identity. Accession numbers for *six2a,* NM_131783; *six2b,* NM_001128734. (b) Amino acid alignments of zebrafish Six2 paralogs with mouse and human Six2. The Six2b protein shares 79% sequence identity and 85-85% similarity with mouse and human Six2. Protein accession numbers for Six2a, NP_571858; Six2b, NP_001122206; mouse Six2, NP_035510; human Six2, NP_058628. Alignments were performed using Jalview [34].

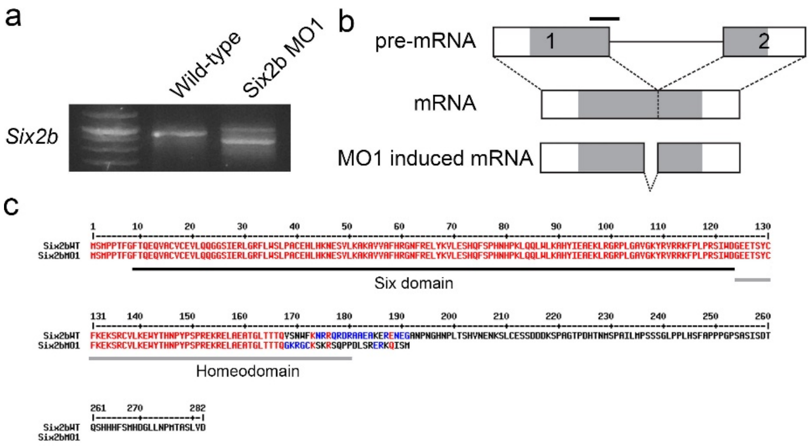

d

	Normal	Phenotype	Total	% phenotype
1ng	41	6	47	13
2ng	32	11	43	26
4ng	20	24	44	55

Supplemental Figure 2: Characterization of *six2b* MO1. (a) PCR amplification of full-length *six2b* in wild-type and MO1 injected embryos. A smaller PCR amplicon is detected along with the wild-type band following MO1 injection corresponding to a deletion from improper splicing. The presence of both bands most likely represents mosaic effect of the MO. Both PCR fragments from MO1 injected embryos were isolated and sequenced confirming the MO1 induced deletion. (b) Schematic of *six2b* splicing in embryos injected with splice donor inhibitor MO1. In the presence of the morpholino, a cryptic splice in exon 1 is utilized resulting in a 59-nucleotide deletion. (c) Amino acid alignment of wild-type and MO1 induced Six2b protein. Injection of MO1 is predicted to result in a protein that has a disrupted homeodomain and missing C-terminus. (d) MO1 injection series at increasing concentrations. Embryos were assessed at 48 hpf using the *wt1a* glomerular marker. Increasing percentage of *wt1a* phenotype was observed with increased MO1 amounts.

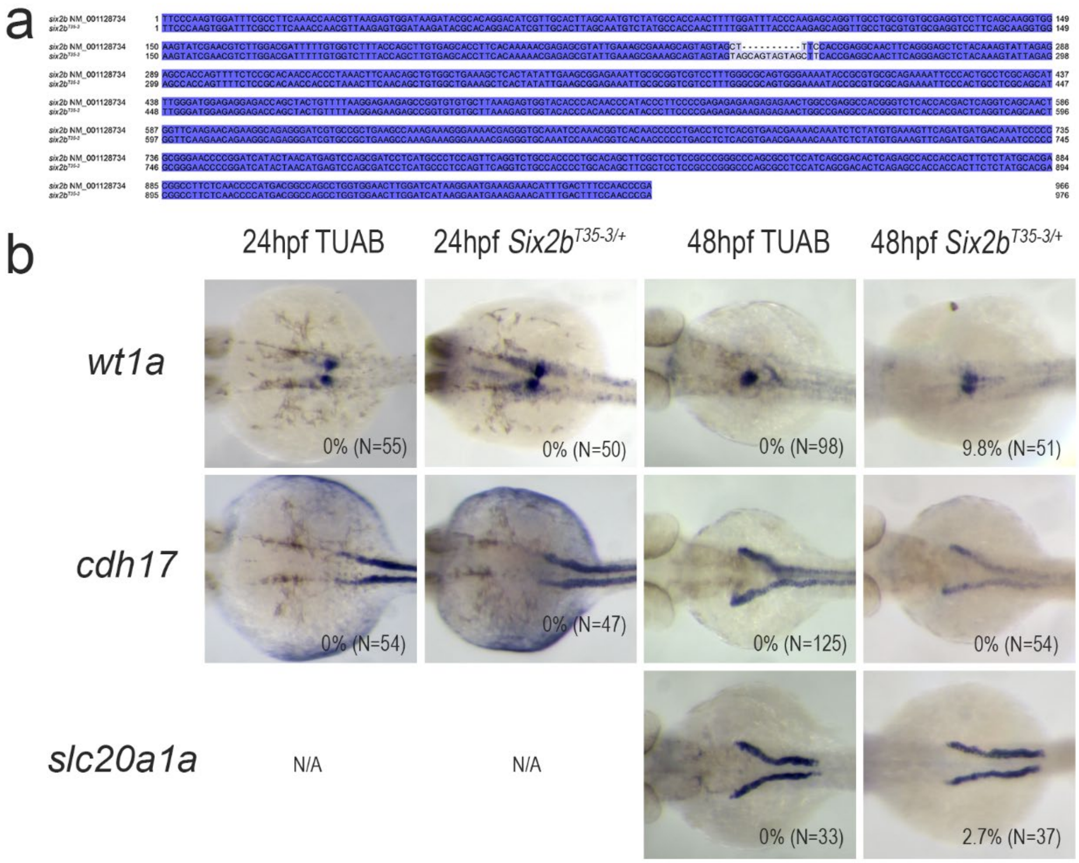

c

	Wild-type	Heterozygote	Homozygote
Observed	14	26	8
Expected	12	24	12

Chi square = 1.833 2 degrees of freedom; two-tailed P = 0.3998

Supplemental Figure 3: Phenotypic analysis of *Six2b*$^{T35\text{-}3/+}$ incrosses. (a) Nucleotide alignment of wild-type *six2b* NM_001128734 with the CRISPR/Cas9 induced mutation *six2b*$^{T35\text{-}3}$ to show the genomic DNA lesion. (b) Six2b mutant heterozygous parents were crossed and resulting embryos were processed for *in situ* hybridization at 24 and 48 hpf. No morphological defects were found at 24 hpf. Some pronephric marker phenotypes were detected at 48 hpf but not at expected frequencies. (c) Chi square analysis of embryos resulting from heterozygous crosses. Genotypes of embryos were at expected frequencies.

Supplemental Table 1: Reagents for *six2b* CRISPR/Cas9 mutagenesis

Target without PAM sequence	GCAGTAGTAGCTTTCCACCG
sgRNA with Sp6 promoter and overlap region	ATTTAGGTGACACTATAGCAGTAGTAGCTTTCCACCGGTTTTAGAGCTAGAAATAGCAAG
Forward primer	5' GCACTTAGCAATGTCTATGCCAC 3'
Reverse primer	5' GGAGAAAACTGGTGGCTCTCTA 3'
Forward primer with T7 sequencing site	5' cacTAATACGACTCACTATAGGGcacGCACTTAGCAATGTCTATGCCAC 3'
